# Progress and Statesmanship

**Published:** 1913-05

**Authors:** 


### Progress and Statesmanship.

Which shall it be—race regeneration or the indiscriminate exercise of man's right to procreation? The lawmakers of our last Legislature answered this question to their evident satisfaction, judging from the dispatch with which they defeated the bills introduced in that body providing for the regulation of marriage and the sterilization of the unfit. The burden of the opposition was stressed on the terribleness of restricting man in his right to procreate his kind. No heed was given to the consequences following the abuse of this privilege on the race, and the question of the right of the unborn child to a heritage of a sound mind and body. And, from the vehemence and levity of the arguments, one might have gathered that to so restrict a man would be to commit the "unpardonable sin." It is true, the highest gift the Creator has bestowed on man and woman is the power to create their kind. But it could scarcely be gainsaid that the purpose of procreation was to maintain the highest type of the race. And if the evolution of the race had been left to natural selection, this object would have been realized. But man, in the exercise of his wonderful free will, has interrupted Nature's way—which usurpation has been responsible for the present condition of society. Now, in this chaotic state, there remains but one thing for him to do, and that is to go a step farther and deliberately control parental selection and the regulation of the offspring.

Speaking of the "unpardonable sin," the only one that Nature knows is bringing into the world a child mentally and physically diseased. Then for a man to exercise his "inalienable right" he must regard the dictates of Nature as more binding than the mere gratification of a passion.

It was Lord Beaconsfield who said: "The public health is the foundation on which repose the happiness of the people and the power of a country. The care of the public health is the first duty of a statesman." Have our legislators performed their duty when they fail to correct conditions that are responsible for our large army of hobos, criminals and prostitutes; that increases the number of insane, idiots and imbeciles, and that denies the degenerate and unfit protection from the curse of transmitting his stigmata to his offspring?

We cheerfully accord to every man the right to express his candid opinion on any question, the same as we hold for ourselves. But this is an era of rapid progress, and there is less excuse for being ill informed today than there was a decade ago, and there should be a greater unanimity of opinion, at least, on the great questions that so vitally affect the race. It seems, though an impossible task to shake some people from old notions. For example, all the arguments and illustrations in the world would not convince some people, among whom are some of our most distinguished Senators, that sterilization, as now advocated, is not the old mutilating operation of castration, and that by such procedure the individual is not deprived of his "inalienable rights." It is one of those psychological anomalies that has its only analogy in another sex-mystery, based on the obscene knowledge unguarded youth gathers in his tender years. These licentious visions are so deeply impressed on his memory that they cannot be effaced. They become obsession, like the "damned spot" in "Macbeth," to appear and reappear to haunt the memory forever.

"If you knew the children's bodies would be paralyzed or pained,
If you knew that God's creation had been robbed of half its rights,
Would you still continue acting as if Nature forgave your faults,
And pretended that she heeds not when we break each natural law?"

